# Sediments from Arctic Tide-Water Glaciers Remove Coastal Marine Viruses and Delay Host Infection

**DOI:** 10.3390/v11020123

**Published:** 2019-01-30

**Authors:** Douwe S. Maat, Maarten A. Prins, Corina P. D. Brussaard

**Affiliations:** 1 Department of Marine Microbiology and Biogeochemistry, NIOZ Royal Netherlands Institute for Sea Research, and University of Utrecht, P.O. Box 59, 1790 AB Den Burg, Texel, The Netherlands; 2 Department of Earth Sciences, Faculty of Science, Vrije Universiteit Amsterdam, 1081 HV Amsterdam, The Netherlands; m.a.prins@vu.nl

**Keywords:** Arctic virus, algae, phytoplankton, sediment, glacier, virus adsorption, infection

## Abstract

Over the past few decades, the Arctic region has been strongly affected by global warming, leading to increased sea surface temperatures and melting of land and sea ice. Marine terminating (tide-water) glaciers are expected to show higher melting and calving rates, with an increase in the input of fine sediment particles in the coastal marine environment. We experimentally investigated whether marine viruses, which drive microbial interactions and biogeochemical cycling are removed from the water column through adsorption to glacier-delivered fine sediments. Ecologically relevant concentrations of 30, 100 and 200 mg·L^−1^ sediments were added to filtered lysates of 3 cultured algal viruses and to a natural marine bacterial virus community. Total virus removal increased with sediment concentration whereby the removal rate depended on the virus used (up to 88% for an Arctic algal virus), suggesting a different interaction strength with the sediment. Moreover, we observed that the adsorption of viruses to sediment is a reversible process, and that desorbed viruses are still able to infect their respective hosts. Nonetheless, the addition of sediment to infection experiments with the Arctic prasinovirus MpoV-45T substantially delayed host lysis and the production of progeny viruses. We demonstrate that glacier-derived fine sediments have the potency to alter virus availability and consequently, host population dynamics.

## 1. Introduction

Coastal marine systems in the Arctic typically contain high concentrations of suspended sediment particles which enter the water column via melting and calving of tide-water glaciers, and via streams and rivers that originate from melting land ice (glaciers) and snow. Glacier-derived sediments contain especially high concentrations of fine clay and silt particles (resp. <8 μm and 8–63 μm), and are produced by abrasion (glacial grinding) of the underlying bedrock. They can remain suspended in the water column for a very long time and extend far off the coast [1,2]. This so-called glacier milk (the fine sediments a.k.a. “rock flour” color the water milky white) can affect the marine food web by reducing phytoplankton photosynthesis due to increased turbidity, and reducing the efficiency of copepod food uptake due to sediment ingestion [1,2,3,4]. Since sediment delivery by the melting and calving of tide-water glaciers is driven by seawater temperature (SST) it shows seasonal dynamics, with most sediment input at the time of highest phytoplankton production and food web activity [2,5,6]. Furthermore, global warming leads to higher annual SST in the Arctic (a predicted 0.03–0.05 °C per year in the 21st century) and subsequently higher melting and calving rates and longer melting and calving seasons, which results in higher total sediment input for a longer time [5,7].

Marine microbial populations are strongly controlled by lytic viruses resulting ultimately in the death of these unicellular hosts [8,9,10,11]; this is also the case in the Arctic [12,13,14,15,16]. Upon the release of virus progeny, the host cells typically undergo cell lysis, which stimulates the microbial loop (bacterial decomposition of the released organic matter [17,18]). Virus production depends on host growth regulating environmental variables, whereas variables that affect virus removal control successful infection (see review by Mojica and Brussaard [19]). Suspended sediments have been found to adsorb viruses, whereby the efficiency depends on the type of viruses, sediment (size, isoelectric point), ionic strength, and pH [20,21,22], hypothetically decreasing their chance to infect a host cell, especially if the sediment settles down the water column [23,24]. Most of the studies to date on sediment-virus interactions are of epidemiological nature, as sediments have been found to be able to remove enteric viruses from, e.g., eutrophic estuaries, fresh water systems and aquifers [25,26,27]. Consequently, the focus of those studies has been on specific types and concentrations of viruses and sediments that apply to those systems or to the systems that may be used to purify those waters, however, they may not necessarily be applicable to most natural marine pelagic environments. For instance, the sediment concentrations used to experimentally remove viruses are typically in the order of magnitude of 1 g·L^−1^ and higher, for example in [22,26]. Such extremely high concentrations are, however, only found in e.g., estuaries with very high river discharge or during storms [2,4]. Typical coastal suspended sediment concentrations are 5 to 1000 times lower in the range of 1–200 mg·L^−1^ e.g., [2,28]. As far as we know, only a few studies have investigated the effects of sediments on aquatic viruses in a more ecological setting, and with inconclusive results [23,29,30]. Wells and Deming [30] did not observe any loss of a phage 9A, which infects the Arctic heterotrophic bacterium *Colwellia psychrerythraea*, with ambient clay concentrations of 10 mg·L^−1^. Hewson and Fuhrman [23] did observe virus removal in natural seawater after the addition of 50 mg·L^−1^ of cleaned and dried sediment from the same site. However, these losses may be influenced by the presence of dead cells and organic matter, including transparent exopolymeric particles (TEP) as the samples were not filtered. Finally, Drewes et al. [29] described a higher virus to prokaryote ratio, and thus possibly lower virus losses in a clear alpine lake not influenced by glaciers, as compared to three similar lakes with high glacier-derived suspended sediment concentrations. More research is needed to obtain a better understanding of the ecological relevance of virus removal by sediments, and with that, the impact on microbial host populations.

The focus of this study was to investigate whether: (i) the loss of viruses by adsorption to (settling) sediments could potentially take place in tide-water glacier influenced marine waters, and (ii) whether the sediments reduce the impact of the viruses on their phytoplankton hosts. The removal (i) was tested by in vitro “adsorption-settling” experiments whereby sediment was added to virus suspensions in glass tubes and allowed to sink out while the concentrations of viruses at the surface of the tubes were monitored. Hence, this simulated the natural export of sediment with adsorbed viruses from surface waters. The possible effects of sediments on the actual interaction of viruses with their phytoplankton hosts (ii) were investigated with one-step and two-step infection experiments, with and without the presence of glacier-derived sediment (at ecologically relevant concentrations), by which the speed of lysis and production of new virus progeny was monitored. The sediments were collected from inner Kongsfjorden, a tide-water glacier influenced fjord system in North Western Svalbard (79° N). Fresh virus lysates of temperate and polar virus cultures, as well as a community of natural temperate viruses (53° N) were used.

## 2. Materials and Methods

### 2.1. Viruses for the Experiments

The adsorption-settling experiments were carried out with 3 different phytoplankton viruses (NIOZ culture collection; Table 1) and a natural virus community (NVC) from the coastal North Sea (sampled at the time of the experiments). All phytoplankton viruses were nucleocytoplasmic large DNA viruses (NCLDV) with particle sizes of 120, 125 and 150 nm for MpoV-45T, MpV-08T and PgV-07T infecting *Micromonas polaris*, *M. commoda* and *Phaeocystis globosa*. These lytic phytoplankton viruses were maintained by fortnightly inoculation of an exponentially growing axenic host culture with 0.2 μm filtered (Minisart polyethersulfone membrane filter, Sartorius A.G., Goettingen, Germany) lysate. Algal cultures were maintained on Mix-TX medium [14], at 4 °C (*M. polaris*) and 15 °C (*M. commoda* and *P. globosa*) and under 80 μmol quanta m^−2^·s^−1^ (ULM-500, Walz, Effeltrich, Germany) at a light:dark cycle of 16:8 (18W/965 OSRAM daylight spectrum fluorescent tubes; München, Germany). Before experimental use, the phytoplankton viruses were filtered through 0.2 μm Minisart polyethersulfone membrane filters (Sartorius A.G., Goettingen, Germany) and diluted to ecologically relevant abundances of 0.5–1 × 10^5^ mL^−1^ [31,32]. By producing the viruses on their hosts under optimal conditions, the highest % of infective viruses (80–100%, obtained by end-point dilution) was assured [33,34].

### 2.2. Sediment Collection for the Experiments

**Figure 1:**
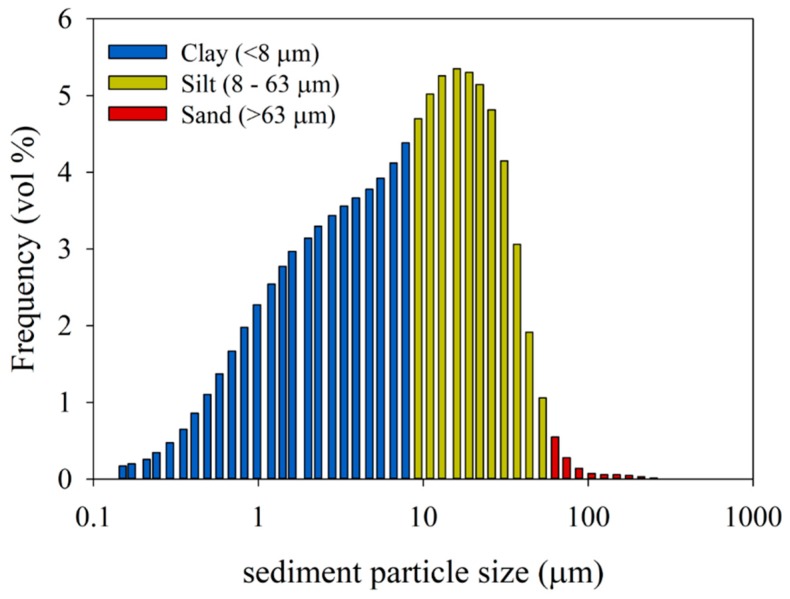
Size distribution of glacier-derived sediment that was used for the adsorption-removal experiments. The *y*-axis shows the relative abundance (vol%) of the particles of the different sediment size classes (µm), which are depicted on the *x*-axis.

Sediment material used for the experiments was collected from the inner fjord in Kongsfjorden, Svalbard in 2009, during a R/V Lance cruise NP09-13 at multicore station NP09-13-26. A Fritsch A22 laser-diffraction particle size analyzer (VU Amsterdam) was used to measure the grain-size distributions of samples obtained from multicore NP08-16-63 (length 41 cm, sampled at 2-cm resolution, *n* = 21) taken at the same location in 2008 (R/V Lance cruise NP08-16). Sample preparation and calculation of clay:silt:sand content follows [37]). The average grain-size distribution of those samples (Figure 1) shows the fine-grained character of the glacier-supplied fjord sediments (clay 43%, silt 46%, sand 1%). A part of the larger sediment particles settled to the bottom of the tubes quite rapidly after addition, observed as formation of precipitation at the bottom of the tube. The finer particles remained longer in the virus suspension. Six hours after mixing (T6), the suspensions were still turbid, whereas they were completely optically clear at T36.

### 2.3. Adsorption-Settling Experiments

Adsorption and removal of viruses with different sediment concentrations was tested by sediment-settling experiments for which 0, 30, 100 or 200 mg·L^−1^ final concentration sediment was added to 10 mL virus suspension in triplicate glass tubes (15 mm diameter). The sediment was pre-weighed and added as a 1 mL suspension in Mix-TX medium (the controls without sediment received only Mix-TX). Right before sediment addition, samples for viral abundances were taken from the tubes as a control for the effects of dilution and handling (T0 h). After sediment addition, the tubes were mixed by gently turning the tube upside down once after covering the tube with parafilm. Samples were kept stable with minimal movement so the sediment with and without viruses could sink out for 36 h (incubation conditions were similar to culturing). As soon as possible (approximately 10 min after sediment addition; T0.15 h), virus samples were taken from the upper half of the tube to investigate the initial loss upon sediment addition. Additional surface samples were taken after 6 and 36 h (T6 and T36). The samples for flow cytometric virus enumeration were directly fixed with 25% glutaraldehyde (EM grade; Sigma-Aldrich, St. Louis, MO, USA) to a final concentration of 0.5%, placed in the fridge at 4 °C for 30 min, flash frozen in liquid nitrogen and stored at −80 °C until analysis [38]. For ecological relevance, the experiments were carried out with viral abundances of 0.5 to 10 × 10^5^ mL^−1^ for the lysates of the algal viruses and approximately 1 × 10^8^ mL^−1^ for the natural virus communities (in situ abundances). To test the maximum removal capacity of the sediment, an additional test was done with higher algal viral abundances (approximately 10^8^ mL^−1^ versus the diluted ones of 0.5 to 10 × 10^5^ mL^−1^ in the diluted tests).

We chose the adsorption-settling method described above as it was found to be the most reproducible compared to centrifugation filtration as a method for determining adsorption rates of viruses to sediment. In short, sediment was added to the lysate in 50 mL Erlenmeyer flasks and mixed well. A sample of 2 mL was then either centrifuged (5 min; 200× *g*) or filtered (0.2 µm syringe filter; Sartopore Midicap, Sartorius A.G., Goettingen, Germany) to remove sediment. The supernatant or filtrate containing the remaining viruses was then fixed for flow cytometry as described above. Mixing and sampling was repeated at T6 and T36 h. In contrast to the settling approach, centrifugation and especially filtration resulted in substantially higher loss of viruses from the controls (i.e., 0–20%, 15–30% and 60–95%, respectively). Although the differences between the controls and sediment treatments were still measurable for the centrifugation experiments, the reproducibility was lower than with the original method. Furthermore, this approach has more ecological relevance, i.e., in a sediment-influenced water column, adsorbed viruses are expected to sink down with the sediment out of the photic zone.

### 2.4. Effects of Detritus and Other Viruses on the Interaction between Sediment and MpoV-45T

An additional experiment was done with the Arctic phytoplankton virus MpoV-45T to test whether sediment pre-exposed to detritus or other viruses (before addition to the MpoV-45T lysate) can occupy potential binding sites and consequently reduce the adsorption of MpoV-45T. PgV lysate (5 × 10^8^ viruses mL^−1^) produced from a dense *Phaeocystis globosa* culture (1 × 10^6^ cells mL^−1^) was added to 4 g·L^−1^ sediment The high density of the lysate assured saturating abundances of viruses and cell debris from the lysed cells. The lysate with added sediment was split into 2 batches, of which one batch (“detritus only”) was sonicated (MSE Soniprep 150, UK) for 3 × 60 s at the highest amplitude to destroy all viruses. For the other batch (“detritus + viruses”) the viruses were left intact. The sediment suspensions were then centrifuged at 3500× *g* for 5 min, after which the supernatant was removed. The sediment was washed once again with medium and centrifuged in the same way, after which the sediment was resuspended in medium (such that a working stock of 2 g·L^−1^ sediment was obtained) and added to undiluted MpoV-45T lysates for the adsorption-settling experiments with final concentrations of 200 mg·L^−1^.

### 2.5. Determination of Virus Removal Rates

The obtained viral abundances were corrected for potential loss in the control treatment (no sediment) after which they were used to calculate the absolute and relative losses of viruses at each time point and the loss rates over time between the time points. Each virus loss calculation for a specific time point was corrected with the abundances of the control treatment at that time point, and each calculation per time interval was corrected for loss in the controls over the specified time period. Because the adsorption dynamics seemed to follow exponential decay (Supplementary Figure S1), specific exponential virus loss rates between 2 time points were calculated and depicted as the constant *λ* of the exponential decay calculations $N\left(t\right)=N\left(0\right)e^{-\lambda t}$, in which *N*(*t*) and *N*(0) respectively stand for the number of viruses at the second and first time point and t stands for the past time in days. Statistics were calculated in the program Sigmaplot^TM^ 14 (Systat software Inc., Chicago Il, USA). Differences between the treatments were tested with one-way ANOVA and Holm-Šídák pairwise comparisons. Significant differences (*p* = 0.05) with the control are depicted in the tables and graphs.

### 2.6. Virus-Host Infection Experiments

The effect of sediment on virus-host interaction was tested with two different experiments: (i) a one-step infection experiment (14:1 virus:host ratio) with 200 mg·L^−1^ final concentration sediment added to determine the virus growth characteristics, and (ii) a two-step infection experiment (0.5:1 virus:host ratio) with 30 and 200 mg·L^−1^ sediment added to mimic more natural conditions and allow testing of the potentially interfering effect of sediments on virus-to-host adsorption. The virus:host ratios were calculated after quantification of the total viruses and algae by flow cytometry.

For the one-step infection experiments, sediment suspension (200 mg·L^−1^ final concentration) was added either to the host directly after the addition of lysate or first to the virus lysate prior to the addition to the host culture (for optimal adsorption to the sediment). For the two-step infection experiment, the sediment was added to the host directly after lysate addition. At the day of the experiments, triplicate 100 mL Erlenmeyer flasks with 50 mL exponentially growing algal culture were inoculated with 0.2 μm filtered (Minisart polyethersulfone membrane filter, Sartorius A.G., Goettingen, Germany) virus lysate. Samples for algae and viruses from these flasks, and from uninfected control cultures (with Mix-TX instead of virus lysate) were taken at T0 (between 5 and 10 min after lysate/sediment addition) and subsequently at regular time intervals until the cultures were visibly lysed (cleared). Cultures were gently mixed every 8 h. Phytoplankton samples (1 mL) were fixed with 0.5% final concentration formaldehyde (Sigma-Aldrich, St. Louis, MO, USA) and virus samples (0.5 mL) with 0.5% final volume of glutaraldehyde (EM grade; Sigma-Aldrich, St. Louis, MO, USA). Samples were fixed for 30 min at 4 °C, followed by flash freezing in liquid nitrogen and storage at −80 °C until analysis. The differences in viral abundances between treatments were tested with one-way ANOVA and Holm-Šídák pairwise comparisons as described above.

### 2.7. Sediment as a Transport Vector for Viruses

To test whether sediment-attached viruses can still infect a host after desorption from sediment, sediment was added to 50 mL fresh 0.2 μm filtered lysates of MpV-08T, PgV-07T and MpoV-45T, and left for 24 h in which the lysate was gently mixed 3 times. The lysate with sediment was then gently filtered over a 47 mm GF/F filter (Whatmann, Maidstone, UK; nominal pore size 0.7 μm) and rinsed 3 times with 2.5 mL Mix-TX medium. The filter was placed in an Erlenmeyer flask, covered with 5 mL Mix-TX and vortexed to resuspend the sediment. Samples were taken for viral abundances and the resultant sediment suspension was added to the respective host cultures in 10:1, 1:10 and 1:100 dilutions in final concentrations of 30 and 200 mg·L^−1^. The obtained abundances may, however, be a slight underestimation of the total amount of potentially desorbed viruses, as a small fraction is expected to have passed through the filters during washing. For the (non-infected) control cultures, the same protocol was used but with sediment that was not exposed to viruses. Lysis was monitored as optical clearance of the cultures in comparison with the controls.

### 2.8. Flow Cytometric Quantification of Viral and Phytoplankton Abundances

Thawed virus samples were 100× to 10,000× diluted in TE buffer (pH 8.2), stained with a final concentration of 0.5 × 10^−4^ of the SYBRGreen I stock (Life Technologies Ltd., Paisley, UK) for 10 min at 80 °C and then quantified on a BD FACSCalibur with the trigger on green fluorescence [38]. The virus data were analyzed with FCS express 5 (De Novo Software, Glendale, CA, USA). Sediment particles did not affect the flow cytometric virus quantification (Supplementary Figure S2). Thawed algal samples were analyzed by flow cytometry using a BD Accuri^TM^ C6 cytometer (BD Biosciences, San Jose, CA, USA), and triggered a red autofluorescence on chlorophyll [39]. Algal data were analyzed directly on the Accuri^TM^ C6 software.

## 3. Results

### 3.1. Adsorption-Settling Experiments

**Figure 2:**
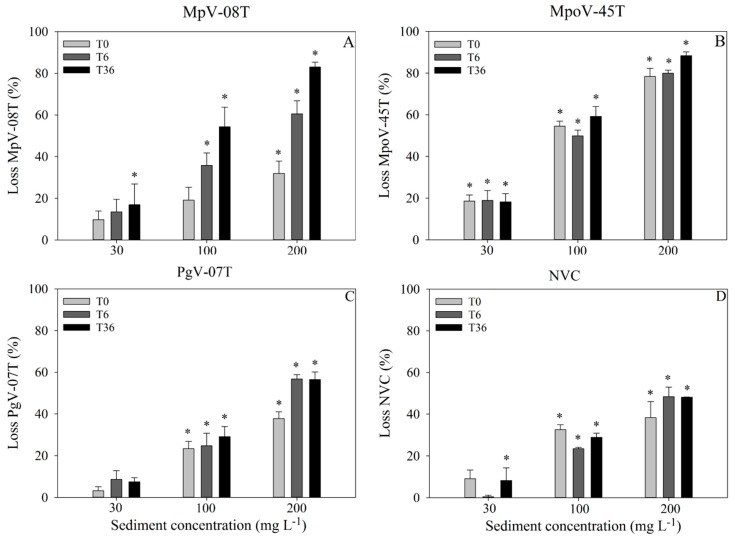
Relative loss of total viruses (%; mean ± s.d.) compared to the control (no sediment) at T0.15 h, T6 h and T36 h, for MpV-08T (**A**), MpoV-45T (**B**), PgV-07T (**C**) and NVC (**D**) and with final sediment concentrations of 30, 100 and 200 mg·L^−1^. Asterisks (*) above the bars show which treatments are significantly different (*p* < 0.05) from the control.

For all virus types, the abundance before the addition of the sediment suspension (T0) did not significantly differ between the treatment tubes (Supplementary Figure S3). Sediment addition led to loss of free viruses for all virus types, with the extent depending on sediment load (Figure 2; Supplementary Table S1). Except for MpV-08T, the highest losses were observed within 15 min after sediment addition (66–100% of the total losses; Figure 2). The losses of MpV-08T were more gradual over time (Figure 2). The Arctic MpoV-45T displayed the strongest initial loss (loss at T0), with up to 19, 55 and 78% of the free viruses lost within 15 min after addition of 30, 100 and 200 mg·L^−1^ sediment, respectively (Figure 2). In general, virus loss increased with sediment load, whereby the 200 mg·L^−1^ treatment showed 15–50% higher losses than the 100 mg·L^−1^ treatment. The 30 mg·L^−1^ sediment treatments showed the lowest losses at the slowest rate (Figure 2). The exponential loss rates in the first 15 min after 200 mg·L^−1^ sediment addition ranged between 247 d^−1^ for MpoV-45T to 62–78 for the other viruses tested. The corresponding virus half-life times were between 23–4 min (Supplementary Table S1).

Despite the differences in viral abundances at the start of the experiment (appr. 10^6^ and 10^5^ mL^−1^ for MpoV-45T and MpV-08T, respectively), the total percentage of viruses lost over the 36 h incubation (Figure 2) was quite similar for both *Micromomas* viruses (18, 59 and 88% for MpoV-45T and 17, 54 and 83% for MpV-08T with 30, 100 and 200 mg·L^−1^ sediment). Likewise, PgV-07T and NVC showed similar total relative losses (7, 29 and 57% and 8, 29, and 48%, respectively), whereas the starting abundances were respectively 10^5^ and 10^8^ mL^−1^. The effect of higher virus concentrations on the removal capacity of the sediment was specifically tested using undiluted algal virus lysates with starting abundances of 0.1–2.4 × 10^8^ mL^−1^. Absolute total losses (0.4–2.2 × 10^7^ viruses mL^−1^; Supplementary Table S2) were of similar magnitude as for NVC. It should be noted that the undiluted MpV-08T had 10 times lower starting abundances than MpoV-45T and Pgv-07T but still sediment addition resulted in comparable total absolute virus losses. This suggests that the maximum binding capacity of the sediment was reached at these virus to sediment ratios, and that our experiments with diluted lysates were not biased by saturated adsorption (Supplementary Figure S4).

**Figure 3:**
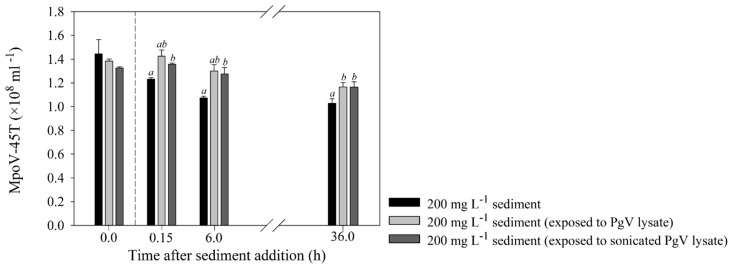
Mean (±s.d.) viral abundances over time, during the 200 mg·L^−1^ adsorption-removal experiments of undiluted MpoV-45T, with clean sediment and sediment that was exposed to either untreated or sonicated PgV-07T lysate to test the influence of detritus on virus removal. The abundances right before sediment addition (T0 h) are depicted on the left side of the vertical dotted line. Further sampling was done at T0, T6 and T36 h. The letters in the graphs above the bars show a significant difference with the control (a) or the 200 mg·L^−1^ treatment without detritus (lysate; b).

The additional experiment testing the effect of lysate/cell detritus exposure of the sediment clearly showed mitigated adsorption of MpoV-45T compared to the standard non-treated sediment treatment (Figure 3). Virus loss was observed only after 36 h, and to a significantly lesser extent than the standard sediment treatment (*p* = 0.007).

### 3.2. Virus–Host Interaction Experiments

**Figure 4:**
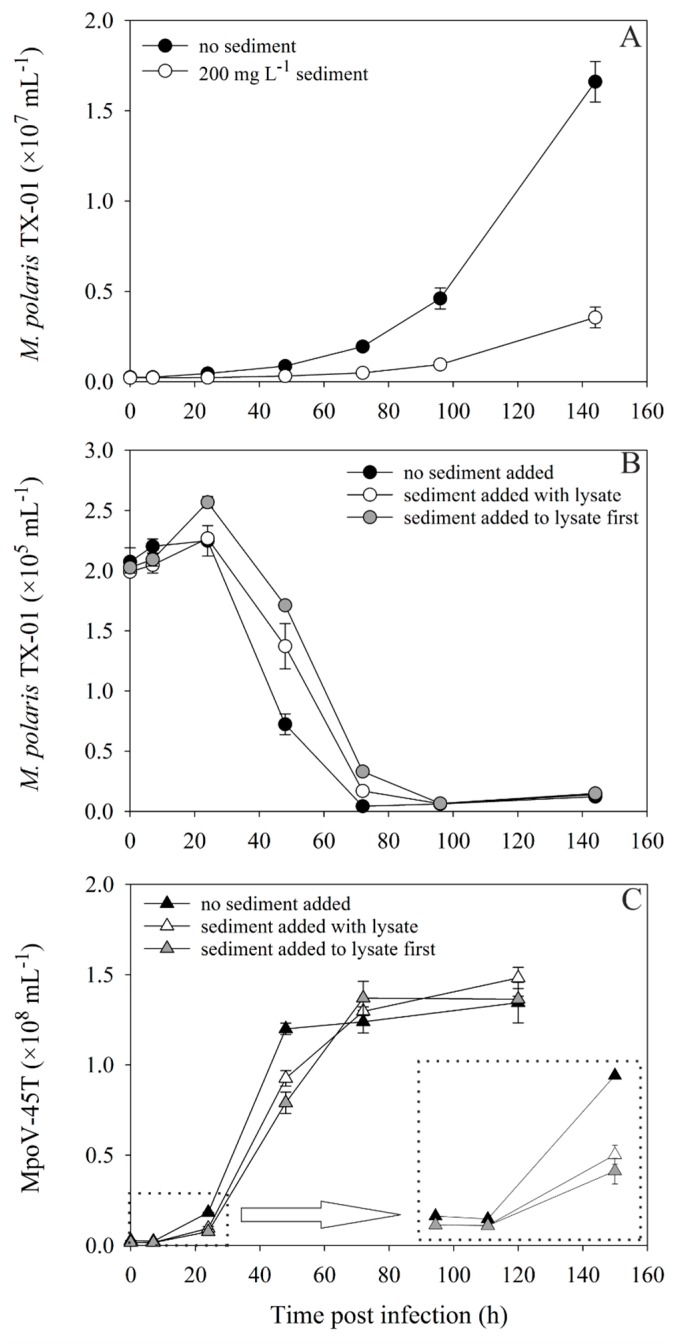
Abundances over time of uninfected (**A**) and infected (**B**) *M. polaris* TX-01, and the virus MpoV-45T (**C**) with and without 200 mg·L^−1^ sediment, during a one-step infection experiment (virus:host ratio = 10:1). The “no sediment” treatments are depicted with black symbols. Sediment was either added to the cultures at the same time as the lysate (“with lysate treatment”; white symbols) or mixed with the lysate first and then added to the host culture (“added to lysate first treatment”; grey symbols). Algae are depicted with circles and viruses with triangles. The inlay in panel C shows the viral abundances in the first 24 h in detail.

The one-step infection experiment to test the direct effects of sediment on virus-host interaction, showed that 200 mg·L^−1^ sediment addition strongly affected the growth of the *Micromonas polaris* TX-01 (Figure 4A). The virally induced lysis of TX-01 and the production of MpoV-45T progeny was clearly delayed for both virus treatments compared to the control (Figure 4B,C). Although the viral abundances were instantaneously reduced after sediment addition (from 2.7 to 1.7 × 10^6^ mL^−1^), the starting virus:host ratio was still 9:1, thus, all hosts could still be infected. Sediment addition did not affect the maximal number of viruses produced (1.4 ± 0.06 × 10^7^ mL^−1^) and consequently the virus burst sizes were comparable (590 ± 54, 665 ± 38 and 552 ± 37 for the “no sediment”, “sediment with lysate” and “sediment first” treatment, respectively). These findings were independent of the *Micromonas* host and virus used, as equivalent results were obtained for an experiment in which *M. commoda* LAC38 (grown at 3 °C) was infected with MpoV-44T, with and without 50 mg·L^−1^ kaolinite clay addition (Supplementary Figure S5). Host lysis and initial virus progeny production were delayed, but the virus burst sizes were the same: 504 ± 29 and 437 ± 90 (*p* = 0.422). The addition of the same type and concentration of sediment to a filtered lysate of MpoV-44T led to relatively gradual losses of up to 50% of total free viruses over time.

**Figure 5:**
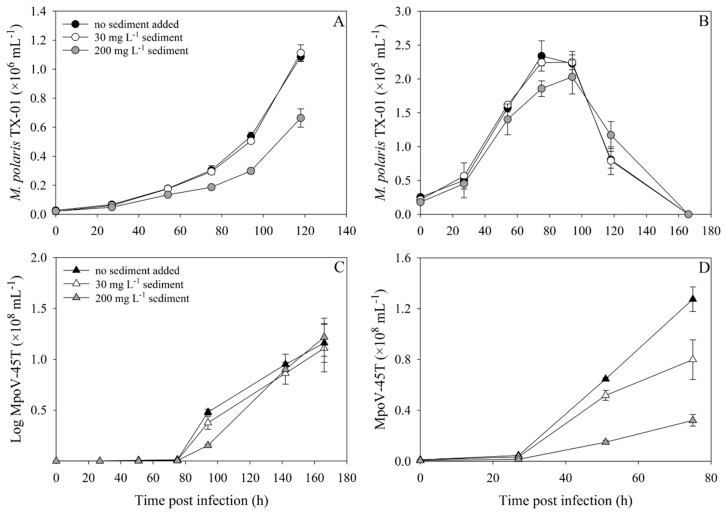
Abundances over time of uninfected (**A**) and infected (**B**) *M. polaris* TX-01, and the virus MpoV-45T (**C**,**D**) with and without 30 or 200 mg·L^−1^ sediment, during a two-step infection experiment (virus:host ratio = 0.5:1). Black, white and grey symbols represent 0, 30 and 100 mg·L^−1^ sediment, with algae as circles and viruses as triangles. Panel D represents a detail of panel C, displaying the viral abundances during the first 75 h.

In the two-step infection experiment, with more ecologically relevant abundances and lower virus:host ratios (0.5:1), the growth of the uninfected *M. polaris* TX-01 control culture was also strongly reduced by the addition of 200 mg·L^−1^ sediment (Figure 5A). However, the 30 mg·L^−1^ cultures were not affected in their growth, compared to the controls without sediment. The 0 and 30 mg·L^−1^ cultures started lysing at least 24 h earlier than the 200 mg·L^−1^ culture (Figure 5B). Lysis for all cultures clearly occurred as a two-step infection cycle, with increasing delay of the virus production rate with increasing sediment concentration (Figure 5C, Supplementary Figure S6). While sediment addition of 200 mg·L^−1^ reduced the viral abundances in the beginning of the experiment, decreasing the virus:host ratio to 0.25:1, the addition of 30 mg·L^−1^ had no significant effect on the virus:host ratio, but still delayed virus production (Figure 5D). Analogous to the one-step infection experiment, the total viruses produced at the end of the experiment was not affected by sediment addition.

### 3.3. Infection of a Host after Desorption from Sediment

Approximately 2 h after resuspension of the virus-exposed and rinsed sediment in culturing medium, a significant proportion of the viruses from the sediment was countable by flow cytometry (0.9, 2.4 and 0.1 × 10^5^ mL^−1^ for MpV-08T, MpoV-45T and PgV-07T, respectively). Within a week, all 3 virus suspensions led to lysis of their respective host (optical clearance of the cultures) at all tested dilutions, showing that at least a fraction of the viruses was released and still infective. The controls with sediment that was not exposed to viruses showed normal growth.

## 4. Discussion

### 4.1. Adsorption-Settling Experiments

Melting tide-water glaciers release large amounts of sediment into Arctic coastal waters, which remain suspended in the water column for extended periods, affecting microbial interactions in the pelagic food web [1,2]. Our data showed that in an experimental setting, suspended glacier-derived sediments removed considerable amounts of algal and bacterial viruses: about 5–90%, depending on the type of virus and sediment concentration used. Whereas previous studies showed that viruses are removed by high experimental sediment concentrations [24,27], we demonstrated that this holds for a simulated Arctic marine ecological setting with relevant types and concentrations of both sediment and viruses. The exact mechanisms of virus removal in this study are not clear. Studies have demonstrated that binding between viruses and sediments can occur due to electrostatic interaction, van der Waals binding, hydrophobic interactions or a combination of all of these, with the contributions of the different mechanisms depending on variables such as the type of virus (size, isoelectric point), type of sediment (size, mineralogy) and chemical composition of the medium in which they are suspended [20,22,24]. Nonetheless, viruses aggregated with the sediment and sank to the bottom of the tubes. In Arctic coastal waters, glacier-derived sediment may thus be an important factor in virus losses, transporting viruses out of the photic zone, and releasing the virus mortality pressure on their hosts. Besides regular seasonal fluctuations, sediment load may be continuous in sediment-rich coastal Arctic waters, gradually decreasing with distance from the tide-water glacier, however, it may also be quite instantaneous and local due to storm-induced water mixing or floating bergy-bits surrounded by a plume of sediment [4,39]. The small grain size of our collected and used sediments demonstrates the spatial relevance as finer particles remain suspended for a longer time and can thus extend further off-coast.

Virus losses increased with higher sediment concentrations and were especially high (up to 88%) with 100 and 200 mg·L^−1^ sediment. These concentrations represent, e.g., the water column within 1 or 2 km distance form a glacier in a summer situation, although the concentration can occasionally even be a lot higher (up to 4 g·L^−1^) during storms [2,4]. Concentrations between 20 and 50 mg·L^−1^ extend many kilometers off the coast, and thus, have a larger zone of influence [2,40]. Our observed virus losses of up to 18% (at ecologically relevant abundances) even under these relatively low sediment concentrations indicate that there will still be a strong impact on food web dynamics in a wide area of the coastal zone.

The increased losses with increased sediment concentration must be the result of increased contact rates between sediment particles and viruses [41]. This is further supported by the increasing total loss with viral starting abundances. The maximum binding capacity of the added sediment for the algal viruses was reached at approximately 1.1 × 10^8^ viruses mg^−1^ sediment. Hewson and Fuhrman [23] showed a 6-fold lower saturation in the adsorption of natural marine virus communities with 1.7 × 10^7^ viruses mg^−1^ sediment. The difference is possibly explained by the type of sediment used. Hewson and Fuhrman [23] only used sediment of 20 µm diameter, whereas in our study most particles were of smaller size with consequently larger surface to volume ratios and potential binding capacity. Moreover, the mineral composition and resulting binding properties may have had an influence as well [20,22], and the possible occurrence of organic matter in their unfiltered samples could have reduced the binding capacity of the sediment ([42,43], our data).

The viruses we used showed differences in removal dynamics, i.e., the Arctic virus MpoV-45T showed the most instantaneous and highest total loss, whereas MpV-08T showed the slowest and PgV-07T the lowest total loss in viruses. Since the sediments used were the same for all viruses and the algal viruses were similar in particle size, it is likely that the viruses differ from each other in characteristics such as hydrophobicity or isoelectric point, resulting in different binding strengths or only binding to a specific type (size class, mineralogical composition) of the sediment particles [20]. The cause of the pronounced removal of the Arctic virus is unclear and remains to be investigated. On the same note it would be interesting to examine whether the mineralogy of sediment, which depends on bedrock geology and geographical region, leads to different virus adsorption dynamics. The reason that NVC achieved a 2–3 times higher total loss with similar starting abundances may be due to the difference in the shape of the virus particle. All NVC viruses were considered bacteriophages, which are typically tailed viruses of smaller size than algal viruses [44], leading to different interaction dynamics with particles [25,41].

### 4.2. Effects of Organic Matter

Based on earlier studies, we hypothesized that added organic matter would occupy potential binding sites for viruses on the sediment, and thus reduce virus adsorption [42,43]. Indeed, organic matter addition from a sonicated PgV-07T lysate mitigated MpoV-45T removal by 200 mg·L^−1^ sediment addition by 31% (i.e., 5% whereas this was 16% without the addition of organic matter). Theoretically, organic matter may thus counter virus losses by glacier-derived sediments in the natural pelagic system. However, for preparation of the detritus-treated sediment we used high concentrations of host cells, resulting in very high organic matter concentrations (final concentrations were the equivalent of 5 × 10^6^ *P. globosa* cells, i.e., 50 μg୼·mL^−1^ [45]). Under natural conditions with lower organic matter concentrations, a reduced mitigation impact can be expected, but to what extent will depend on local conditions such as, e.g., high lysis during the demise of phytoplankton blooms or varying organic matter concentrations directly supplied by glaciers [2,46]. A better understanding of this process is warranted as viruses attached to organic matter such as TEP will be (temporarily) unable to infect a new host [19,47], further complicating the interaction effects between sediments, organic matter, viruses and their hosts.

### 4.3. Virus–Host Interaction

Sediment addition delayed not only the growth of the algal host (*M. polaris* TX-01 as well as *M. commoda* LAC38), but also the production of the respective lytic viruses, MpoV-45T and MpoV-44T. The reduced growth of the host after 200 mg·L^−1^ sediment was added seems to be the logical result of increased turbidity, and the consequent reduced light availability [34,48,49]. However, light intensities measured inside the culture were not significantly different from the no-sediment treatment (79 ± 7, 81 ± 5 and 69 ± 9 μmol quanta m^−2^·s^−1^ for 0, 30 and 200 mg·L^−1^ sediment, respectively). Furthermore, the photosynthetic efficiency (Fv/Fm; Water-PAM fluorometry, Walz, Effeltrich, Germany) of *M. pusilla* TX-01 was not affected by sediment treatment, indicating that photophysiology was not affected by turbidity. Microscopic observations revealed that some of the *M. polaris* TX-01 cells were attached to sediment particles on the bottom of the chamber, but this was not enough to explain the difference in abundances between the 0 and 200 mg·L^−1^ sediment treatment. We speculate that possibly a milder mechanical effect due to collision with particles may have caused the reduction in growth rate [50,51]. Wolfe et al. [52] showed how phytoplankton cells respond to mechanical stress by reduced growth and DMS production.

In the one-step infection experiments, lysis of the host was slightly delayed by the addition of sediment (200 mg·L^−1^ glacier-derived sediment or 50 mg·L^−1^ kaolinite clay), which may have resulted from the sediment-induced stress of the host cell. Alternatively, anti-virus effects of elevated DMS concentrations [52,53] may have reduced the ratio of infectious virus to susceptible host cell, thereby negatively influencing the proliferation efficiency of the viruses [54]. The experiment with more ecologically relevant concentrations of viruses (the two-step infection experiments) demonstrated that even at lower virus to host ratios, the addition of sediment negatively affected host growth (200 mg·L^−1^) and virus production (both 30 and 200 mg·L^−1^). These experiments indicate that removal of viruses by sediment can strongly reduce the chance of successful infection and virus proliferation.

Although the total number of produced viruses did not vary amongst the treatments, the observed slower production can be expected to have ecological consequences in the natural environment. Furthermore, continuous input and settling of glacier-derived sediment particles [2] will cause persistent removal of viruses, and consequently, will further reduce the chances of infecting a new host. Conversely, we also showed that the phytoplankton viruses adsorbed to glacier sediment (including the Arctic MpoV-45T) were able to desorb while maintaining their infectivity. Sediment particles may thus act as a vector of vertical and horizontal transport of viruses [26,55,56], and subsequently, they have the potential to influence host population dynamics and spread infection (assuming a suitable host is available). In summary, our study demonstrates that fine-grained suspended sediments released from melting glaciers impact both virus removal and production.

## 5. Conclusions

We found that glacier-derived sediments led to substantial losses of algal and bacterial viruses, and that the losses increased with higher sediment load and viral abundance. Organic matter prevented virus removal, but the ultimate effect on host-virus dynamics is likely to be limited as the attached viruses will still be restricted in successfully encountering their host. Sediment delayed host lysis and virus production, also under ecologically relevant conditions. Our study shows that glacier derived sediments have the potential to strongly impact microbial interactions by reducing host mortality through diminished virus infection as well as declined host growth. Continued global warming is anticipated to further enhance virus loss by adsorption to glacier melt-induced sediments. Additionally, melt-induced vertical stratification and the consequent reduction in mixing may accelerate settling of sediments with viruses attached away from suitable hosts. These findings indicate the need for more direct in situ investigations of the effects of glacier-derived fine sediments on virus removal and viral lysis.

## Figures and Tables

**Table 1:** Specifics of the viruses used in the adsorption experiments: viral strains or communities used, origin of isolation, temperature for culturing and experiments, host organisms, references of viral strains. ^1^ formerly known as *M. pusilla*. ^2^ mainly prokaryotes.

Virus Used	Origin	Temp. (°C)	Host	Reference
MpV-08T	Temperate	15	*Micromonas commoda* LAC38 ^1^	[35]
MpoV-45T	Arctic	4	*Micromonas polaris* TX-01	[14]
PgV-07T	Temperate	15	*Phaeocystis globosa* G(A)	[36]
NVC	Temperate	15	Natural community ^2^	

## Supplementary Materials

The following are available online at http://www.mdpi.com/1999-4915/11/2/123/s1. Supplementary Materials: Table S1: Calculated absolute and relative losses and exponential loss rates, Table S2: Absolute and relative total virus losses of the undiluted lysates, Figure S1: Exponential loss of MpoV-45T to sediment, Figure S2: Flow cytometry scatter plots of the viruses used with and without sediment, Figure S3: Absolute viral abundances over time during the adsorption-removal experiments, Figure S4: Virus losses under different starting abundances and sediment concentrations, Figure S5: The interaction of MpoV-44T with kaolinite clay, Figure S6: Log viruses over time during the two-step infection experiment.
