# Supplementary material for: Sediments from Arctic Tide-Water Glaciers Remove Coastal Marine Viruses and Delay Host Infection
Source: Viruses. 2019 Jan 30;11(2):123. doi: 10.3390/v11020123 (PMC6409924; doi:10.3390/v11020123)
Supplement: Supplementary file 1 [file viruses-11-00123-s001.pdf]

**Supplement Table S1:** Calculated absolute and relative losses and exponential loss rates with 30, 100 and 200 mg $L^{-1}$ sediment from the adsorption-removal experiments with MpV-08T, MpoV-45T, PgV-07T and the natural virus community (NVC). Values significantly different from the controls are in italics (p=0.05). The exponential loss rates (only calculated for significant decreases) were calculated for 3 time frames: i) -0.15 – 0h, ii) 0 – 6h, and 6 – 36h and only significant values are shown. *only for upper frame (absolute numbers).

| | | **Sediment ($mg\ L^{-1}$)** | **MpV-08T ($\times 10^{4}$)*** | **MpoV-45T ($\times 10^{4}$)*** | **PgV-07T ($\times 10^{4}$)*** | **NVC ($\times 10^{7}$)*** |
|---|---|---|---|---|---|---|
| **Virus loss (viruses $mL^{-1}$)** | T0 | 30 | 0.53 ±0.20 | *1.3 ±0.2* | 0.14 ±0.08 | 1.0 ±0.5 |
| | | 100 | 1.0 ±0.3 | *4.0 ±0.2* | *1.0 ±0.2* | *3.7 ±0.3* |
| | | 200 | *1.7 ±0.3* | *5.7 ±0.3* | *1.7 ±0.1* | *4.4 ±0.9* |
| | T6 | 30 | 0.75 ±0.3 | *1.4 ±0.4* | 0.40 ±0.20 | 0.05 ±0.01 |
| | | 100 | *2.0 ±0.3* | *3.6 ±0.2* | *1.2 ±0.3* | *2.6 ±0.06* |
| | | 200 | *3.4 ±0.4* | *5.8 ±0.1* | *2.6 ±0.1* | *5.4 ±0.5* |
| | T36 | 30 | *0.99 ±0.06* | *1.2 ±0.3* | 0.34 ±0.09 | 1.0 ±0.8 |
| | | 100 | *3.2 ± 0.6* | *4.0 ±0.3* | *1.3 ±0.2* | *3.5 ±0.3* |
| | | 200 | *4.9 ±0.1* | *6.0 ±0.1* | *2.6 ±0.2* | *5.9 ±0.1* |
| **Virus loss (% of total)** | T0 | 30 | 9.7 ±4 | *19 ± 3* | 3 ±2 | 9 ±4 |
| | | 100 | *19 ±6* | *55 ±2* | *23 ±4* | *33 ±2* |
| | | 200 | *32 ±6* | *78 ±4* | *38 ±3* | *38 ±8* |
| | T6 | 30 | 14 ±6 | *19 ±5* | 9 ±4 | 0.44 ±0.80 |
| | | 100 | *36 ±6* | *50 ±3* | *25 ±6* | *23 ±1* |
| | | 200 | *60 ±6* | *80 ±1* | *57 ±2* | *48 ±5* |
| | T36 | 30 | *17 ± 1* | *18 ±4* | 7 ±2 | 8 ±6 |
| | | 100 | *54 ±9* | *59 ±5* | *29 ±5* | *29 ±2* |
| | | 200 | *83 ±2* | *88 ±2* | *57 ±4* | *48 ±0.1* |
| **Exponential loss rate ($d^{-1}$)** | -0.15 – 0h | 30 | - | 33 ±6 | - | - |
| | | 100 | - | 126 ±54 | 43 ±16 | 63 ±23 |
| | | 200 | 62 ±16 | 247±55 | 76 ±19 | 78 ±24 |
| | 0 – 6h | 30 | - | - | - | - |
| | | 100 | 0.9 ±0.4 | - | - | - |
| | | 200 | 2.2± 0.3 | - | 1.4 ±0.2 | - |
| | 6 – 36h | 30 | - | - | - | - |
| | | 100 | 0.3 ±0.1 | - | - | - |
| | | 200 | 0.7 ±0.0 | 1.4 ±0.1 | - | - |

**Supplement Table S2:** Absolute and relative total virus losses of the undiluted lysates of MpV-08T, MpoV-45T and PgV-07T in relation to their starting abundances and sediment concentration to test whether higher viral abundances reduce the removal of sediment due to occupation of binding sites.

| **Virus type** | **Starting abundance ($ml^{-1}$)** | **Sediment ($mg\ L^{-1}$)** | **Absolute loss ($\times 10^7\ ml^{-1}$)** |
|---|---|---|---|
| **MpV-08T** | $1.3 \pm 0.15 \times 10^7$ | 30 | 0.44 ±0.07 |
| | | 100 | 1.1 ±0.03 |
| | | 200 | 1.2 ±0.01 |
| **MpoV-45T** | $1.4 \pm 0.18 \times 10^8$ | 30 | 0.49 ±0.10 |
| | | 100 | 1.1 ±0.3 |
| | | 200 | 2.2 ±0.4 |
| **PgV-07T** | $2.4 \pm 0.71 \times 10^8$ | 30 | 0.93 ±0.70 |
| | | 100 | 2.3 ±1.1 |
| | | 200 | 2.0 ±0.8 |

**Supplement Figure S1**

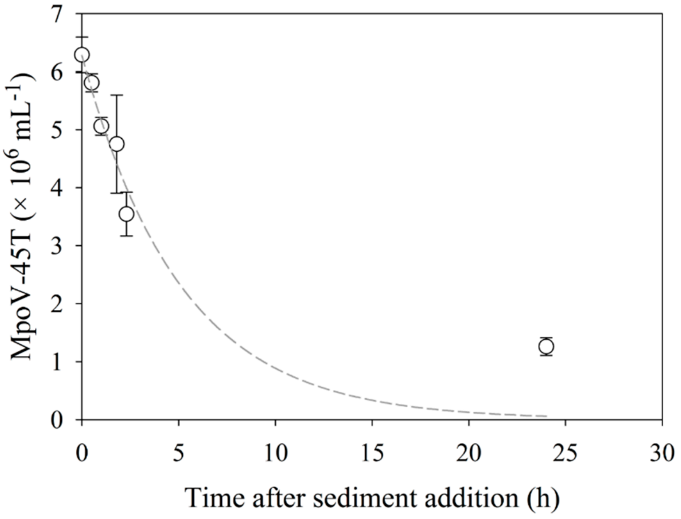


**Supplement Figure S1:** MpoV-45T abundance over time (n=3) exposed to 50 mg $L^{-1}$ montmorillonite clay. The loss of viruses follows an exponential decay process: $N(t) = 6276998\ e^{-0.2t}$ ($r^2$=0.90).

**Supplement Figure S2**

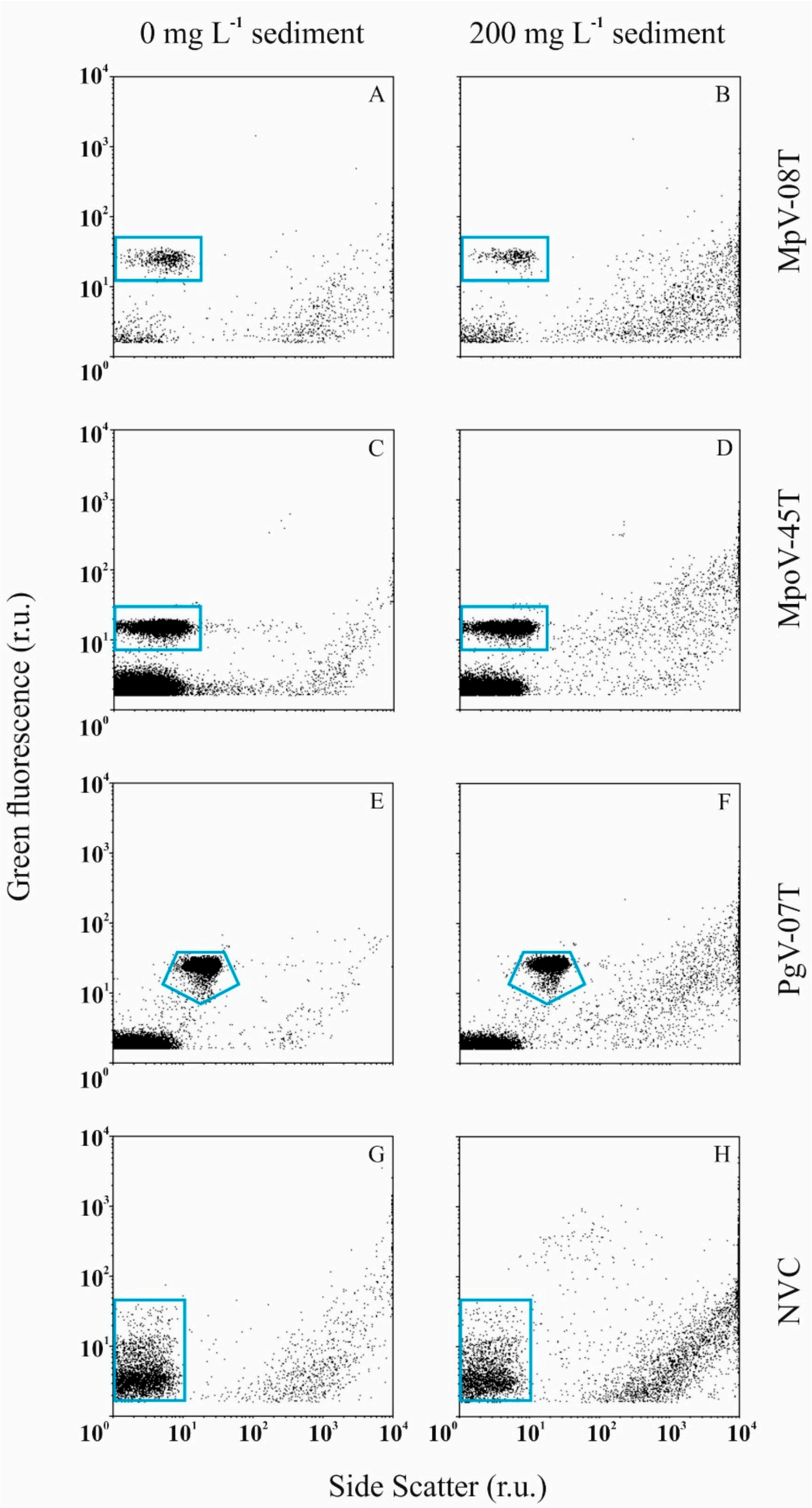


**Supplement Figure S2.** Cytograms of the 3 algal viruses (A-F) and the natural virus community (NVC; G-H) for 0 mg $L^{-1}$ (A,C,E,G) and 200 mg $L^{-1}$ (B,D,F,H) sediment addition treatment. The specific viruses were discriminated based on their green nucleic acid-specific green fluorescence and the side scatter signal (cluster is depicted by blue frame).

**Supplement Figure S3**

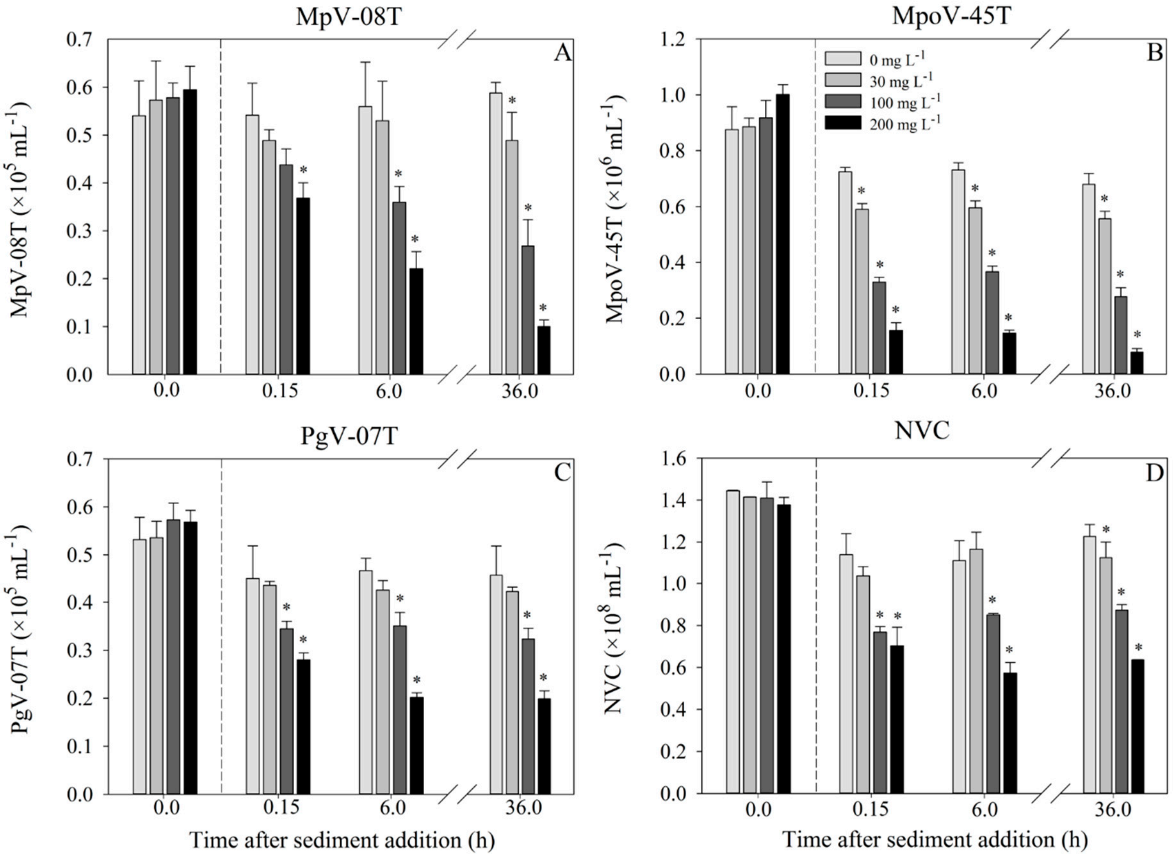


**Supplement Figure S3.** Absolute viral abundances (mean ± s.d.) of MpV-08T (A), MpoV-45T (B), PgV-07T (C) and NVC (D) over time during the adsorption-removal experiments and with 0 (control), 30, 100 and 200 mg $L^{-1}$ glacier-derived sediment. The abundances right before sediment addition (T0 h) are depicted on the left side of the vertical dotted line. Further sampling was done at T0.15 h, T6 h and T36 h. Asterisks (*) above the bars show which treatments are significantly different ($p<0.05$) from the control. A part of the decrease in viral abundances between T0 and T0.15 (visible in the controls) is due to the addition of sediment suspension or (for the controls) medium.

**Supplement Figure S4**

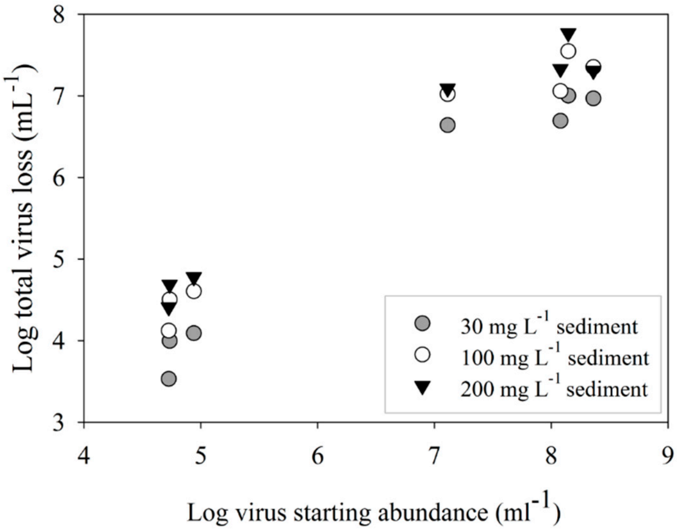


**Supplement Figure S4:** Total virus losses under the different starting abundances of diluted and undiluted MpV-08T, MpoV-45T and NVC and under sediment concentrations of 30, 100 and 200 mg $L^{-1}$ sediment. Note the log scale for both the x- and y-axis.

**Supplement Figure S5**

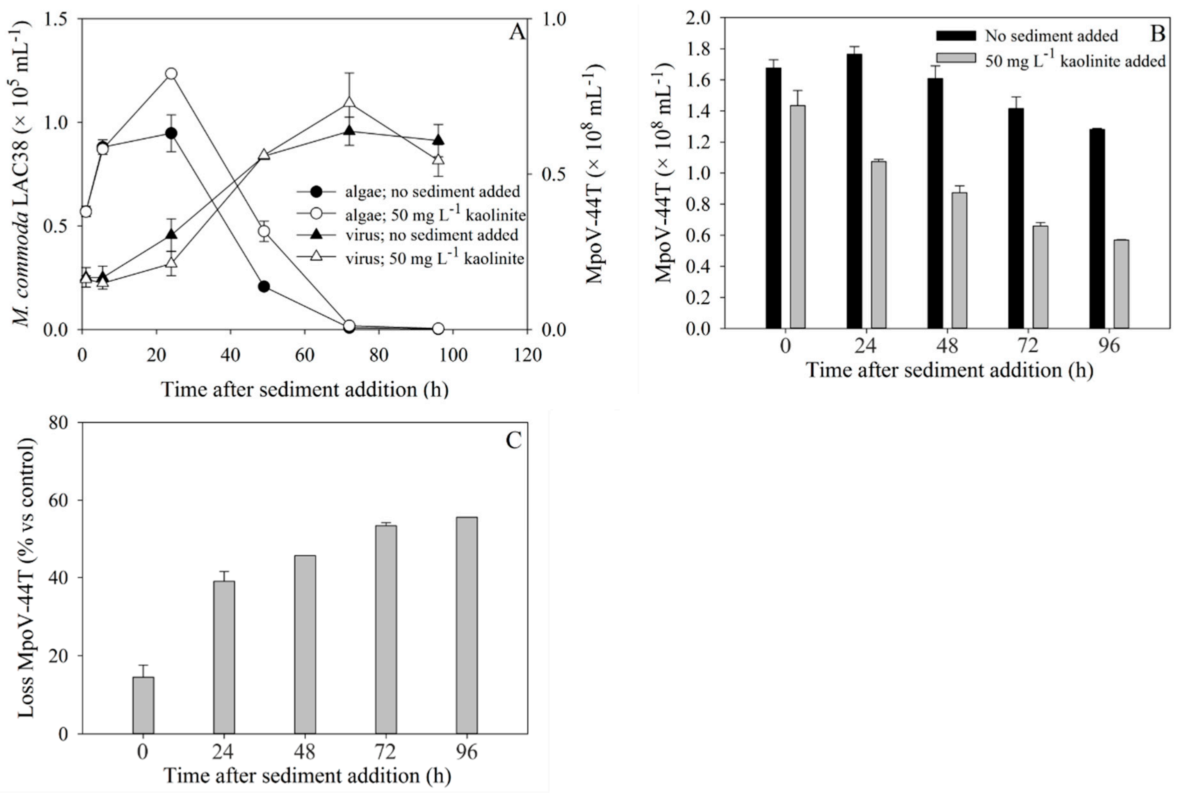


**Supplement Figure S5:** The interaction of MpoV-44T with the clay kaolinite: A) a one-step infection cycle of MpoV-44T (triangles) on *Micromonas commoda* LAC38 (circles) with (white symbols) and without (black symbols) 50 mg $L^{-1}$ kaolinite (temperature 3°C); B) absolute decreases in viral abundances in a filtered lysate with 50 mg $L^{-1}$ kaolinite (grey bars) versus a control without sediment (black bars); C) Virus losses (%) relative to the control without sediment.

**Supplement Figure S6**

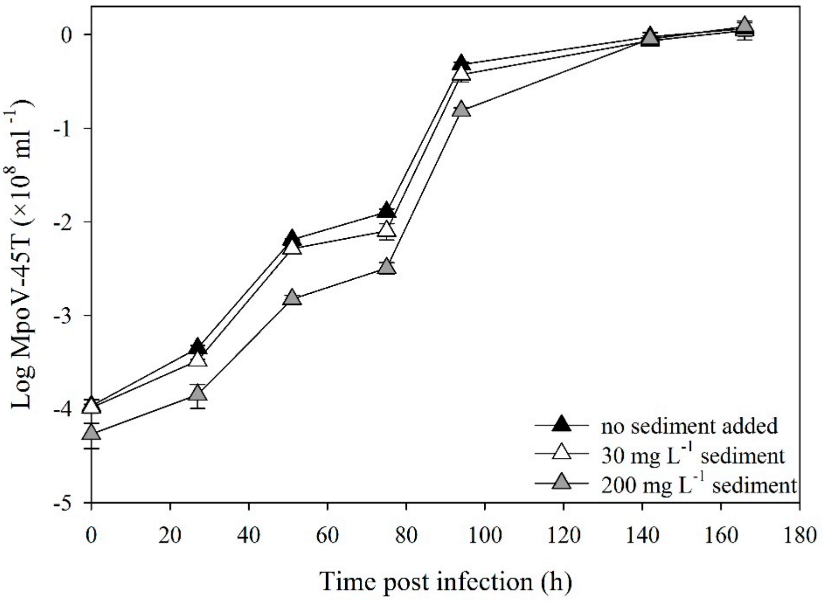


**Supplement Figure S6:** Viruses over time during the two-step infection experiment. The log y-axis clearly shows the two-step nature of the virus-host interaction, i.e. viruses are produced in different two cycles.
